# Knowledge-based planning for intensity-modulated proton therapy of the breast and chest wall with regional lymph nodes: development, validation, and comparative evaluation of two models

**DOI:** 10.3389/fonc.2026.1717884

**Published:** 2026-03-02

**Authors:** Parker Anderson, Yihang Xu, Robert Kaderka, Jonathan Cyriac, Hayden Guerrero, Elizabeth Bossart, Cristiane Takita, Nesrin Dogan

**Affiliations:** Department of Radiation Oncology, University of Miami Miller School of Medicine, Miami, FL, United States

**Keywords:** breast cancer, dosimetry, intensity modulated proton therapy, knowledge based planning, machine learning

## Abstract

**Purpose:**

Knowledge-based planning (KBP) can improve efficiency and consistency in radiotherapy. In intensity-modulated proton therapy (IMPT) for breast cancer, particularly with regional lymph nodes, treatment planning remains complex. This study developed, validated, and compared two KBP models for IMPT of the breast and chest wall (CW) to enhance plan quality, reduce inter-planner variability, and streamline workflows.

**Materials and methods:**

Fifty patients (25 left-sided, 25 right-sided) previously treated with IMPT to the breast or CW including regional lymph nodes were used for model development. A combined model was trained on all patients, while side-specific models were developed separately for left- and right-sided treatments. Planning objectives were refined via iterative replanning of 20 training cases. For validation, 20 additional patients (10 left, 10 right) were evaluated using the respective models. Dosimetric metrics for target volumes and organs at risk (OARs) were compared among model-generated and clinical plans using paired t-tests (*p* < 0.05). An expert physician ranked each plan for clinical preference.

**Results:**

All KBP-generated plans met clinical constraints. Both models achieved target coverage comparable to clinical plans. The combined model produced cooler hot spots in axillary, supraclavicular, and internal mammary nodes, with modest improvements in OAR sparing (heart, esophagus, trachea, thyroid). The side-specific model showed similar benefits, particularly for esophagus and thyroid sparing. Between models, side-specific plans had cooler hot spots, while the combined model offered slightly better OAR sparing. Physician review deemed all KBP plans clinically acceptable, preferring the KBP plan in 11 of 20 cases.

**Conclusions:**

KBP models can generate clinically acceptable IMPT plans for breast and CW with regional lymph nodes, achieving comparable target coverage to clinical plans with modest improvements in OAR sparing and hot spot reduction, supporting their potential to streamline treatment planning.

## Introduction

Current radiation treatment planning methods often rely on time-consuming trial-and-error process that is highly dependent on the planner’s expertise. These limitations can lead to inconsistencies in plan quality and have driven increased interest in automated planning methods. Knowledge-based planning (KBP) is increasingly being adopted in clinical practice to generate plans that are more consistent, objective, and time-efficient. KBP is a data-driven approach to intensity-modulated radiation therapy (IMRT) planning that leverages previously created high-quality plans to inform future ones. By learning from these previous plans, KBP can generate models capable of predicting dose-volume histograms (DVHs) and optimization objectives for new plans (1–4). The inherent objectivity in plans created by KBP models can help reduce subjectivity and improve consistency in the planning process. These planning methods have previously resulted in plans that are clinically comparable, or even better than those achieved with traditional methods across various disease sites (4–6). Due to these acceptable target coverage metrics and improved organ-at-risk (OAR) sparing and reduced optimization times (7–9), the clinical use of KBP continues to expand.

Use of KBP for intensity-modulated proton therapy (IMPT) has also shown promising clinical potential, as KBP models can account for the distinct physical properties of protons when predicting treatment outcomes (10). KBP has been proven to be a valuable starting point for IMPT planning, capable of generating clinically comparable plans with significantly reduced optimization times (5, 11, 12). These reductions in planning time are particularly advantageous in adaptive radiotherapy, where new plans must be created rapidly to accommodate anatomical changes during treatment (13). Previous studies have demonstrated the successful use of KBP for IMPT in various disease sites, including prostate, head-and-neck, liver, and gastroesophageal cancers (5, 14–17).

Although previous studies have demonstrated that KBP models can generate clinically comparable or superior plans for IMRT in breast cancer (18, 19), there are no other studies on the use of KBP for IMPT in breast cancer to our knowledge. This represents a significant gap in the field, given the well-established advantages of IMPT in the treatment of breast cancer (20, 21), its increase in use, and the inherent complexity of IMPT planning. In this study, we aimed to fill this major gap in the research by developing, validating, and comparing the performance of two KBP models for IMPT of breast and chest wall (CW), including regional lymph nodes. The models were designed to accommodate a range of dose prescriptions and anatomical variations, reflecting the clinical diversity of breast cancer cases. Additionally, model-generated plans were compared with manually created plans to assess their clinical acceptability and overall quality.

## Materials and methods

### Patient population

This institutional review board (IRB)-approved study included 50 patients previously treated with IMPT to the whole breast or CW, and regional lymph nodes (25 left-sided and 25 right-sided). An independent cohort of 20 patients was used for validation of the KBP models. A range of dose prescriptions were included to reflect the typical clinical practice in this treatment setting. Clinical target volume (CTV) prescriptions for breast or CW and regional lymph nodes were 40.05Gy in 15 fractions, 45Gy in 25 fractions, 50Gy in 25 fractions, or 50.4Gy in 28 fractions.

### Treatment planning

All plans were generated on a Varian ProBeam (Varian Medical Systems, Palo Alto, CA) pencil beam scanning system. Planning used a 36 × 50 cm field size with proton energies ranging from 70–220 MeV, spot sigma of 4–7 mm, 0.4 cm spot spacing, and a 3 cm or 5 cm range shifter when applicable. Clinical plans were manually generated using robust optimization (Eclipse 16.1), accounting for 5 mm translational error in six directions and ±3.5% proton range uncertainty. This resulted in 12 robustness scenarios per plan, applied across all CTVs to address the intrinsic uncertainties of IMPT (22–24).

For most patients, CTVs were prescribed to receive at least 95% of the prescribed dose to 95% of the volume (V95% ≥ 95%). For internal mammary (IM) lymph nodes, a subset of patients had prescriptions of V90% ≥ 90%.

**Figure 1:**
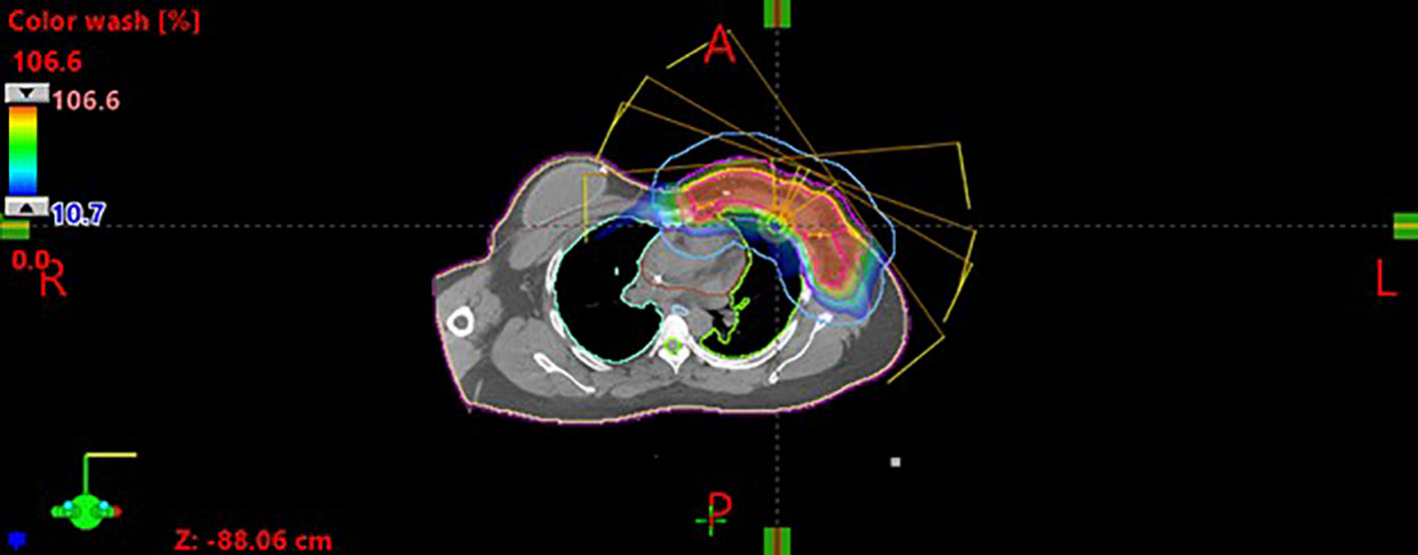
Example patient to show beam arrangement and dose distribution.

Multifield optimization (MFO) was employed for all patients included in this study, typically using three to five en-face beams from variable gantry angles (2–4 between 10 and 70 degrees, and one at 345 degrees.) (see **Figure 1**). Optimization was performed using the nonlinear universal proton optimizer (NUPO 16.1, Eclipse, Varian Medical Systems, Palo Alto, CA), and dose calculations were performed using the proton convolution superposition (PCS 16.1, Eclipse, Varian Medical Systems, Palo Alto, CA) algorithm with a dose grid of 0.2cm x 0.2cm x 0.2cm. Clinical plans were created to meet all relevant planning constraints, as outlined in **Table 1**. However, due to patient-specific anatomical variations, it was not always possible to achieve full target coverage and optimal OAR sparing simultaneously. In such cases, final plan acceptance was based on the clinical judgment of the planner and treating physician.

**Table 1:** Clinical constraints for targets and OARs.

Structures	Constraints
The Union of all CTVs (CTV_All)	D0.03cc < 115%
	V95 > 95%
	V90 (worse case) > 90%
Chest Wall or Breast CTV (CTV_CW)	D0.03cc < 115%
	V95 > 95%
Axilla Lymph Node CTV (CTV_Axilla)	D0.03cc < 110%
	V95 > 95%
Supraclavicular Lymph Node CTV (CTV_SCV)	D0.03cc < 110%
	V95 > 95%
Internal Mammary Lymph Node (CTV_IMN)	D0.03cc < 110%
	V95 > 95%/V90 > 90%
Heart	Dmean < 1Gy
Ipsilateral Lung (Lung_ipsi)	V5Gy < 45%
	V10Gy < 30%
	V20Gy < 20%
Contralateral Lung (Lung_contra)	V4.8Gy < 10%
Contralateral Chest or Breast (CW_contra)	V3Gy < 10%
Esophagus	D0.03cc < 35Gy
Spinal Cord	D0.03cc < 20Gy
Trachea	D0.03cc < 50Gy
Thyroid	Dmean < 30Gy
Ipsilateral Humeral Head (Humeral Head_ipsi)	Dmean < 20Gy
Skin	D0.03cc < 50Gy

Vx represents the relative volume receiving > x% of the prescription dose (e.g., V95) or > x Gy (e.g., V40Gy); D0.03cc represents the maximum dose to any 0.03 cc of the structure (a voxel); Dmean represent the mean dose received by a structure.

### Model training

Two KBP models were created using the commercial KBP software (RapidPlan^PT^ v.16.1, Varian Medical Systems, Palo Alto, CA). The first was a combined model trained on all 50 breast patients (25 left-sided and 25 right-sided). The second was a side-specific model – one for left-sided and one for right-sided disease; each was trained and validated only on cases from the corresponding side and evaluated on same-side validation cases.

Each plan was prescribed to the primary target CTV_All, defined as the Boolean union of all CTVs (CW or breast and each lymph nodes target). For each plan in the training set, a 1-cm ring structure (Ring) was generated around CTV_All to limit hotspot spillage beyond the target boundaries. An additional ring structure (NS_Ring) was created with an interior wall of -1 cm and outer wall of +3 cm from CTV_All.

To establish a baseline for model performance, a subset of 20 patients (10 left-sided and 10 right-sided) was used as a tuning cohort. For each case, plans were generated using the KBP model with the same beam angles, dose prescriptions, and robustness settings as the corresponding clinical plans. Each tuning case included volumes for every relevant OARs to ensure that appropriate optimization objective could be created by the KBP models. During model development, optimization objectives for each volume were either generated automatically or manually defined by the user, providing the baseline for model-driven plan generation. As suggested by previous studies, we tuned the model (i.e., the preset objective/priority template) to align the automatically generated objectives list with our institution’s clinical trade-offs and planning protocol, since objective placement and relative priority assignment are user-preference–dependent and can be overridden (12).

The dose–volume histograms (DVHs) of the model-generated plans were compared to those of the original clinical plans. Tuning was performed through an iterative, trial-and-error process: when model-generated plans failed to meet clinical standards, the optimization objectives were adjusted, and new plans were generated. This process was repeated until the majority of tuning cases produced plans that were clinically comparable to, or better than, the original clinical plans. All optimization objectives were left unchanged during optimization except for the BODY structure. We observed that the initial KBP plans tended to produce increased hot spots. Therefore, during the first optimization run, the BODY objective priority was set to 0 to allow the optimizer to converge without a strong BODY penalty. The plan was then re-optimized in a second iteration after increasing the BODY priority to 1000. This two-step approach helped reduce dose hot spots. A complete list of optimization objectives and their robustness settings is provided in **Table 2**.

**Table 2:** Final optimization objectives used for each model.

Structure	Objective type	Relative volume (%)	Dose	Priority
CTV_All	Upper	0.0	101.5%	250
	Lower*	98.0	101.0%	150
	Lower*	100.0	99.0%	200
CTV_Axilla	Upper	0.0	101.5%	250
	Lower	98.5	101.0%	150
	Lower*	100.0	99.0%	200
CTV_CW	Upper	0.0	101.5%	250
	Lower	98.5	101.0%	150
	Lower*	100.0	99.0%	200
CTV_IMN	Upper	0.0	101.5%	250
	Lower	97.0	99.0%	200
	Lower*	100.0	95.0%	100
CTV_SCV	Upper	0.0	101.5%	250
	Lower	100.0	99.0%	100
	Lower*	100.0	99.0%	100
A_LAD	Upper (fixed vol., generated dose)	0.0	Generated	65
	Mean		3.000 Gy	100
	Mean		Generated	49
	Line (pref. target)	Generated	Generated	60
BODY	Upper	0.0	105.0%	0
BrachialPlexus	Upper	0.0	100.0%	100
	Upper (fixed vol., generated dose)	0.0	Generated	65
	Line (pref. target)	Generated	Generated	50
ChestWall_Contra	Upper	0.0	10.0%	200
	Upper	5.0	3.0%	200
	Upper (fixed vol., generated dose)	0.0	Generated	100
	Line (pref. target)	Generated	Generated	100
Esophagus	Upper	0.0	32.000 Gy	150
	Upper	0.0	35.000 Gy	180
	Upper (fixed vol., generated dose)	0.0	Generated	250
	Mean		Generated	100
	Line (pref. target)	Generated	Generated	100
Heart	Upper	0.0	95.0%	150
	Mean		0.850 Gy	280
	Mean		Generated	150
	Line (pref. target)	Generated	Generated	150
Humerus	Upper	0.0	90.0%	150
	Upper (fixed vol., generated dose)	0.0	Generated	100
	Line (pref. target)	Generated	Generated	80
Lung_Contra	Upper	0.0	5.000 Gy	200
	Upper	0.0	8.000 Gy	250
	Mean		Generated	120
	Mean		0.500 Gy	200
	Line (pref. target)	Generated	Generated	120
Lung_Ipsi	Upper	17.0	15.000 Gy	200
	Upper	0.0	98.0%	150
	Upper	30.0	4.000 Gy	200
	Upper	40.0	2.000 Gy	200
	Mean		Generated	120
	Mean		7.000 Gy	200
	Line (pref. target)	Generated	Generated	120
NS_Ring	Upper (fixed vol., generated dose)	0.0	Generated	100
Ribs	Upper	0.0	99.0%	200
	Upper (fixed vol., generated dose)	0.0	Generated	120
	Line (pref. target)	Generated	Generated	99
Ring	Upper	0.0	101.0%	250
Skin	Upper	0.0	95.0%	200
	Line (pref. target)	Generated	Generated	100
Spinal Cord	Upper	0.0	7.00 Gy	200
	Upper (fixed vol., generated dose)	0.0	Generated	120
	Line (pref. target)	Generated	Generated	99
Thyroid	Mean		32.0%	100
	Mean		Generated	99
	Line (pref. target)	Generated	Generated	80
Trachea	Mean		Generated	150

(*) indicates that robust optimization was enabled for these objectives.

Once acceptable results were achieved for all tuning cases, these finalized optimization objectives were used to generate the final KBP model. Robust optimization was applied to all CTVs, and both models were then created using the same set of optimization objectives determined by this iterative tuning process.

### Model validation

An independent set of 20 patients (10 left-sided and 10 right-sided), not included in the training cohort, were used to validate both KBP models. For each patient, two plans were generated – one using the combined model and one using the side-specific model – and compared against each other as well as the original manually generated clinical plans. Only one iteration of optimization was performed without further changing anything on the objectives list.

Plan performance was evaluated using DVH metrics to evaluate target coverage and OAR sparing, including a selected worst-case robustness scenario for CTV_All. Statistical comparisons between model-generated and clinical plans were performed using paired t-tests, with significance defined as *p* < 0.05.

An expert breast cancer physician was asked to perform a blinded review comparing clinical plans with those generated by each KBP model. For each of the 20 validation patients, the physician evaluated target coverage and OAR sparing, ranking each plan on a 1–3 scale, 1 being the most clinically acceptable, 3 the least.

## Results

**Table 3** shows a comparison of DVH parameters between model-generated plans and their corresponding clinical plans. Plans created by the combined model had no significant differences in target coverage for any dosimetric parameters. On average, plans created with the combined model showed cooler hot spots for target volumes, including the axillary nodes (D0.03cc = -1.55 ± 2.80%), supraclavicular (SCV) nodes (D0.03cc = -2.02 ± 2.79%), and internal mammary (IM) nodes (D0.03cc = -2.97 ± 3.55%). Robustness of these plans was very comparable to clinical plans, as there were no significant differences in coverage in the CTV_All worst-case scenario.

**Table 3:** Comparison of dose-volume indices (mean ± standard deviation) between manually optimized clinical plans (clinical) and KBP model-generated plans (combined and side-specific).

	Clinical	Combined	Side-specific	Combined-Clinical	p-value	Side-specific -Clinical	p-value	Side-specific -Combined	p-value
CTV_All D0.03cc (%)	108.62 ± 2.61	109.02 ± 3.25	108.57 ± 3.33	0.4 ± 3.58	0.62	-0.05 ± 3.74	0.95	-0.45 ± 0.79	0.02
CTV_All V95 (%)	97.42 ± 1.7	97.08 ± 1.31	96.94 ± 1.35	-0.34 ± 1.95	0.45	-0.48 ± 2.1	0.31	-0.15 ± 0.67	0.34
CTV_All V90 (%, worse case)	96.75 ± 1.75	97.28 ± 0.94	97.32 ± 1.01	0.53 ± 1.77	0.20	0.57 ± 1.75	0.16	0.04 ± 0.21	0.40
CTV_CW D0.03cc (%)	108.11 ± 2.66	108.72 ± 3.49	108.34 ± 3.51	0.61 ± 3.51	0.45	0.23 ± 3.61	0.78	-0.38 ± 0.73	0.03
CTV_CW V95(%)	97 ± 2.37	96.44 ± 1.61	96.33 ± 1.56	-0.55 ± 2.58	0.35	-0.66 ± 2.62	0.27	-0.11 ± 0.69	0.48
CTV_Axilla D0.03cc (%)	106.42 ± 2.13	104.87 ± 2.48	104.55 ± 2.33	-1.55 ± 2.8	0.02	-1.87 ± 2.7	0.01	-0.32 ± 0.63	0.04
CTV_Axilla V95 (%)	99.07 ± 1.18	99.58 ± 0.58	99.35 ± 0.67	0.51 ± 1.27	0.09	0.29 ± 1.35	0.35	-0.23 ± 0.67	0.15
CTV_SCV D0.03cc (%)	106.83 ± 2.03	104.81 ± 2.64	104.2 ± 2.55	-2.02 ± 2.79	0.00	-2.63 ± 2.83	0.00	-0.61 ± 1.17	0.03
CTV_SCV V95 (%)	97.56 ± 2.38	96.25 ± 1.85	96.49 ± 1.78	-1.31 ± 3.12	0.08	-1.07 ± 3.03	0.13	0.25 ± 0.7	0.13
CTV_IMN D0.03cc (%)	105.93 ± 3.66	102.95 ± 2.48	102.55 ± 2.28	-2.97 ± 3.55	<0.01	-3.38 ± 3.31	0.00	-0.4 ± 0.77	0.03
CTV_IMN V90 (%)	98.46 ± 2.39	99.05 ± 1.15	99.15 ± 1.2	0.6 ± 2.46	0.24	0.69 ± 2.33	0.27	0.1 ± 0.66	0.59
Heart Dmean (Gy)	0.87 ± 0.43	0.68 ± 0.43	0.67 ± 0.43	-0.19 ± 0.2	<0.01	-0.2 ± 0.19	<0.01	0 ± 0.06	0.72
Lung_ipsi V5Gy (%)	39.29 ± 3.88	38.34 ± 3.41	38.26 ± 3.48	-0.96 ± 4.19	0.32	-1.03 ± 4.51	0.32	-0.07 ± 0.62	0.60
Lung_ipsi V10Gy (%)	28.6 ± 3.6	27.58 ± 2.77	27.42 ± 2.9	-1.02 ± 4.2	0.29	-1.18 ± 4.55	0.26	-0.16 ± 0.7	0.32
Lung_ipsi V20Gy (%)	14.68 ± 2.77	13.88 ± 2.29	13.7 ± 2.36	-0.8 ± 3.09	0.26	-0.99 ± 3.32	0.20	-0.18 ± 0.57	0.17
Lung_contra V4.8Gy (%)	0.97 ± 1.39	1.01 ± 1.56	0.96 ± 1.5	0.04 ± 1.25	0.90	-0.02 ± 1.23	0.95	-0.05 ± 0.13	0.07
CW_contra V3Gy (%)	4.89 ± 4.92	4.51 ± 3.99	4.48 ± 3.95	-0.38 ± 1.69	0.37	-0.42 ± 1.62	0.31	-0.03 ± 0.2	0.49
Esophagus D0.03cc (Gy)	35.97 ± 6.23	31.98 ± 6.63	33.28 ± 5.08	-3.99 ± 5.3	<0.01	-2.69 ± 5.2	0.03	1.3 ± 2.31	0.02
Spinal Cord D0.03cc (Gy)	8.08 ± 4.57	6.52 ± 3.82	6.72 ± 3.85	-1.56 ± 3.49	0.06	-1.36 ± 3.38	0.09	0.2 ± 0.87	0.32
Trachea D0.03cc (Gy)	40.94 ± 8.76	36.5 ± 8.47	39.45 ± 9.01	-4.43 ± 4.47	<0.01	-1.49 ± 4.21	0.21	2.94 ± 1.98	<0.01
Thyroid Dmean (Gy)	21.76 ± 5.32	17.84 ± 3.21	18.65 ± 3.27	-3.93 ± 3.03	<0.01	-3.11 ± 3.06	<0.01	0.81 ± 0.5	<0.01
Humeral Head_ipsi Dmean (Gy)	4.57 ± 3.92	4.68 ± 3.9	4.04 ± 2.27	0.11 ± 1.33	0.82	-0.52 ± 1.99	0.48	-0.63 ± 1.69	0.32
Skin D0.03cc (Gy)	50.04 ± 2.13	50.6 ± 2.33	50.43 ± 2.44	0.56 ± 1.64	0.14	0.39 ± 1.76	0.33	-0.17 ± 0.4	0.08

Comparison was done with a paired t-test (p<0.05 is significant).

Moreover, plans generated by the combined model demonstrated improved sparing of several OARs, including the heart (Dmean= -0.19 ± 0.20 Gy), esophagus (D0.03cc= -3.99 ± 5.30 Gy), trachea (D0.03cc = -4.43 ± 4.47 Gy), and thyroid (Dmean = -3.93 ± 3.03 Gy), as compared to clinical plans. No other differences between the combined model and clinical plans were statistically significant.

Plans produced by the side-specific model also displayed no significant differences in target coverage compared manually generated clinical plans. Significant reductions were observed in the hot spots for the axillary nodes (D0.03cc = -1.87 ± 2.70 Gy), SCV nodes (D0.03cc = -2.63 ± 2.83 Gy), and IM nodes (D0.03cc = -3.38 ± 3.31 Gy). The side-specific model also yielded improved sparing of the esophagus (D0.03cc = -2.69 ± 5.20 Gy) and thyroid (Dmean = -3.11 ± 3.06 Gy). There was no significant difference in coverage in the worst-case coverage scenario, further demonstrating comparable target coverage. All other differences between plans generated by the side-specific model and clinical plans were not statistically significant.

When comparing the side-specific and combined models directly, it was found that there were no significant differences in target coverage for any metric. Notably, the side-specific model resulted in cooler hot spots for every CTV, including CTV_All (D0.03cc = -0.45 ± 0.79 Gy), CW (D0.03cc = -0.38 ± 0.73 Gy), axillary nodes (-0.32 ± 0.63) SCV nodes (D0.03cc = -0.61 ± 1.17 Gy), and IM nodes (D0.03cc = -0.40 ± 0.77 Gy). There was no difference in coverage in the worst-case uncertainty scenario, demonstrating comparable robustness between the two models. Plans created by the combined model had overall improved sparing, specifically for the esophagus (D0.03cc = 1.30 ± 2.31 Gy), trachea (D0.03cc = 2.94 ± 1.98Gy), and thyroid (Dmean = 0.81 ± 0.50 Gy). Overall, the side-specific model had comparable performance to the combined model, with entirely comparable target coverage, improved hot spot doses, and slightly worse OAR sparing results.

In the blinded review of the 20 validation patients, the expert physician preferred the model-generated plans in 11 cases. Among these 11 plans, the combined model was favored over the side-specific model in 7 cases. **Table 4** provides an overview of the physician’s rankings for the three plans: clinical, side-specific KBP, and combined KBP.

**Table 4:** Ranking of clinical, side-specific KBP, and combined KBP plans by a blinded expert physician for 20 validation cases.

Patient	Rank 1	Rank 2	Rank 3
1	Clinical	Side-specific	Combined
2	Clinical	Combined	Side-specific
3	Combined	Side-specific	Clinical
4	Clinical	Side-specific	Combined
5	Combined	Side-specific	Clinical
6	Side-specific	Combined	Clinical
7	Clinical	Combined	Side-specific
8	Clinical	Side-specific	Combined
9	Clinical	Combined	Side-specific
10	Clinical	Combined	Side-specific
11	Combined	Side-specific	Clinical
12	Clinical	Side-specific	Combined
13	Side-specific	Combined	Clinical
14	Clinical	Combined	Side-specific
15	Side-specific	Combined	Clinical
16	Combined	Side-specific	Clinical
17	Combined	Side-specific	Clinical
18	Side-specific	Clinical	Combined
19	Combined	Side-specific	Clinical
20	Combined	Side-specific	Clinical

Rank 1 indicates the most clinically favorable plan, while Rank 3 indicates the least favorable.

**Figure 2:**
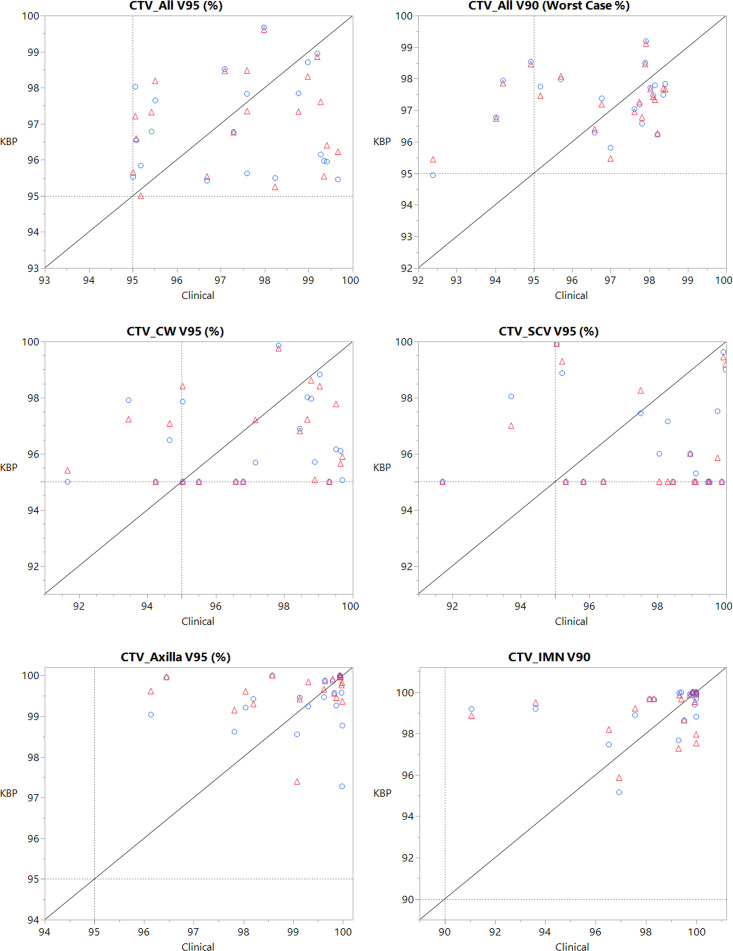
CTV target coverage parameters, where each point is one patient, with the x-coordinate being the clinical plan value and the y-coordinate being the value generated by the KBP plan. Red triangle points represent values created by the combined model, while blue circle points represent values created by the side-specific model. The line represents a y=x unity line. Dashed reference line represents the institutional constraint.

**Figure 3:**
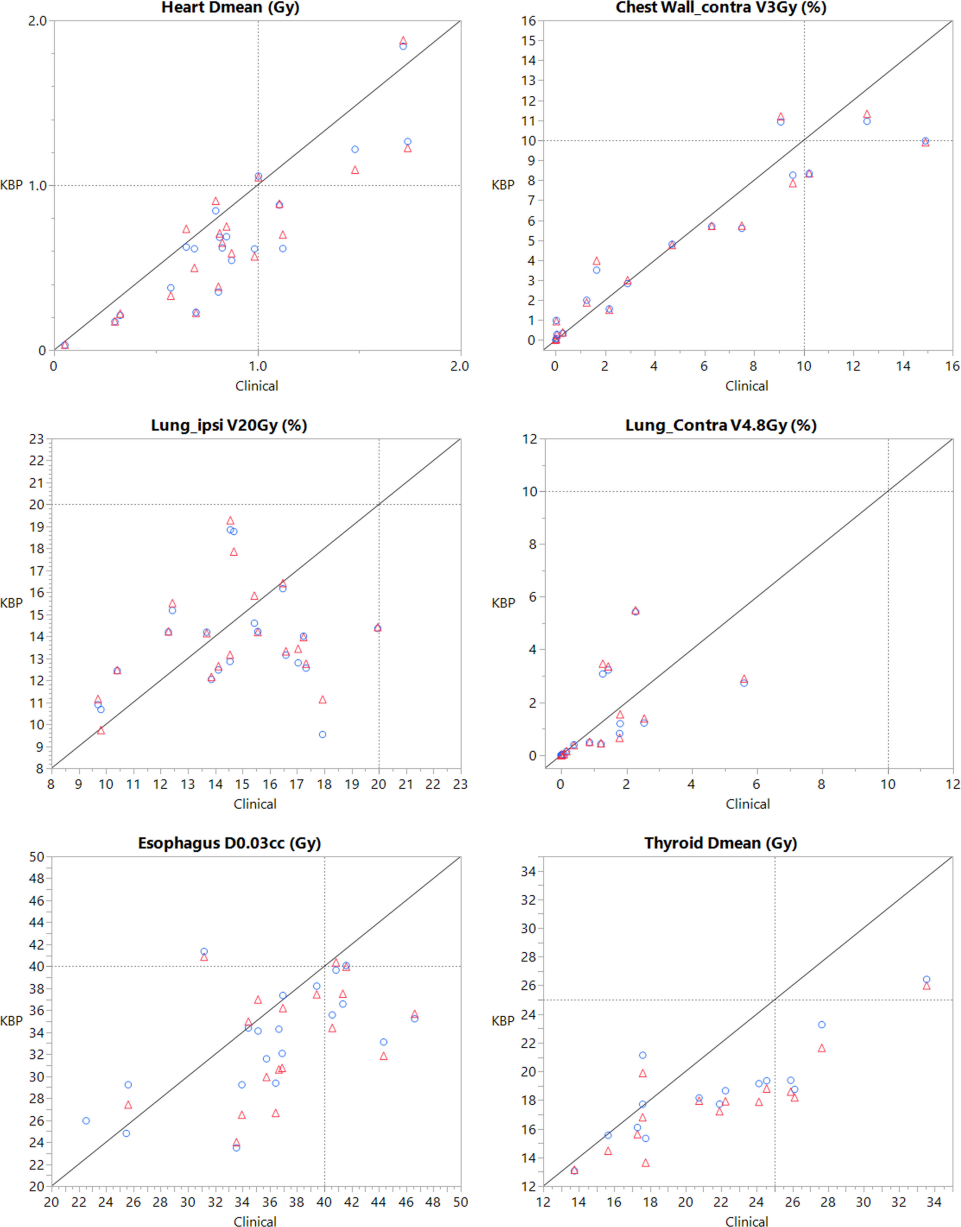
OAR sparing parameter, where each point is one patient, with the x-coordinate being the clinical plan value and the y-coordinate being the value generated by the KBP plan. Red triangle points represent values created by the combined model, while blue circle points represent values created by the side-specific model. The line represents a y=x unity line. Dashed reference line represents the institutional constraint.

**Figure 2** shows case-by-case comparisons of CTV target coverage for both models relative to their respective clinical plans, and **Figure 3** shows comparisons of coverage for relevant OAR parameters. All plans generated by both KBP models were clinically acceptable with respect to overall target coverage and OAR sparing. On average, the robustness of the model-generated plans was comparable to that of the clinical plans.

## Discussion

The primary objective of this study was to develop and compare two KBP models for IMPT of the breast or CW with regional lymph node involvement. Given the limited research available on KBP for breast IMPT, this study also aimed to evaluate model design strategies that could inform the development of future standardized approaches.

Given that breast and CW IMPT are delivered unilaterally, it was initially unclear as to whether developing laterality-specific KBP models or a single combined model would yield better performance. To investigate this, both approaches were implemented and evaluated. All three models demonstrated good fit for OAR DVH estimation, with comparable average χ² values (combined: 1.04 ± 0.03; left: 1.05 ± 0.05; right: 1.10 ± 0.08), where χ² quantifies the difference between the original and estimated DVHs. On average, the combined model had slightly higher hot spots in target volumes and improved OAR sparing. When compared to manually generated clinical plans, both models achieved cooler hot spots with improved OAR sparing. The combined model may be clinically preferable, as it provides comparable target coverage while offering superior OAR sparing, particularly for the esophagus, trachea, and thyroid. An additional practical advantage is that a single combined model allows all patients to be planned consistently without needing to separate cohorts by laterality.

The reason for the differences between the two models is not immediately clear. One possible explanation lies in the iterative tuning process, during which optimization objectives were refined to generate acceptable plans for both left- and right-sided cases. This likely resulted in a more generalized and robust set of objectives, inherently better suited to a model trained on both sides, rather than being narrowly optimized for a single side. As a result, the combined model benefited from training on the full dataset of fifty patients, allowing it to learn from a broader range of anatomical variations and treatment scenarios. In contrast, the side-specific model was limited to only 25 cases per laterality (right- or left side) for training, potentially reducing its ability to generalize and limiting model performance. This effectively reduced the training cohort for each side-specific model to only twenty-five patients, which may have limited its performance during validation. In future studies, increasing the number of training cases for each side, ideally using two side-specific models with at least 50 cases each would provide a more accurate assessment of the potential performance of side-specific models. However, in the present study, it was important to maintain identical cohort sizes across both modeling approaches to ensure a fair and controlled comparison of the two models. Regardless, the effect training cohort size has previously been shown to have little to no effect on plan outcomes (25, 26).

When evaluated independently, all 20 validation plans generated by the combined model were deemed clinically acceptable and comparable to their manually optimized counterparts. Of these 20 cases, 3 technically exceeded the clinical constraint for mean heart dose (Dmean<1Gy). However, further review revealed that this constraint was also not met in the original clinical plans. In these instances, anatomical limitations likely prevented both full target coverage and the heart constraints from being met simultaneously. Consequently, clinical planners prioritized target coverage, accepting an increased heart dose based on clinical judgment. The KBP model appeared to reflect a similar prioritization, favoring CTV coverage at the expense of heart sparing. Despite exceeding the heart constraint, the model-generated plans still demonstrated improved heart sparing compared to their clinical counterparts and were thus considered clinically comparable.

Plans generated by the combined model were, on average, clinically comparable, if not superior, to their manually optimized counterparts. On average, there were no significant differences in target coverage for any of the dosimetric parameters. Most plans had cooler hot spots in the axillary, SCV, and IM nodes. Importantly, the combined model consistently achieved significantly better OAR sparing, specifically for the heart, esophagus, trachea, and thyroid. These OAR structures are particularly important to protect in breast and CW IMPT cases. There was also no significant difference in the CTV_All worst-case coverage scenario, demonstrating clinically comparable robustness in plans created by the combined model. The benefits of improved OAR sparing, combined with entirely comparable target coverage and robustness, support the overall clinical utility of the combined model.

Compared to manually generated plans, those created using the side-specific model demonstrated clinically comparable, if not superior, performance for most metrics. Notably, there were cooler hot spots in the axillary, SCV, and IM nodes. Combined model plans also showed improved sparing for several OARs, including the heart, esophagus, and thyroid. These OAR constraints are highly relevant in IMPT of breast and chest wall, so this improved sparing is very desirable in the planning process.

The results of the blinded physician review further support the conclusion that the model-generated plans were highly comparable to manually generated plans. A model-generated plan was selected as superior for 11 of the 20 patients. Factors contributing to the preference for model-generated plans included improved target coverage, enhanced heart sparing, and reduced hot spot doses within target volumes. It is likely that alternative model objectives could have produced even more favorable plans. For standardized comparison, each validation plan was normalized to meet minimum coverage criteria for all targets. While the models were trained using broadly applicable objectives to enhance generalizability, clinical preferences can vary across reviewers and institutions; therefore, objective sets and weights can be fine-tuned to reflect local practice priorities.

The results of this study are consistent with previous studies on IMRT, rather than IMPT, for breast cancer. Wang et al. developed a left-sided KBP model in which all plans met prescription requirements, with comparable or improved OAR sparing (18). Similarly, Fogliata et al. incorporated both single and bilateral breast volumetric arc therapy (VMAT) patients into a single KBP model, resulting in plans that were generally comparable or superior to manually generated plans (27). To our knowledge, no prior study has directly compared the performance of two breast/CW cancer models trained on the same patient cohort, as done here. Our findings indicate that KBP is a valuable tool for IMPT planning of breast and chest wall cancers. We recommend creating combined models that include both left- and right-sided patients, as this approach appears to provide better planning outcomes. Nonetheless, side-specific models (left- or right-sided only) remain clinically viable alternatives.

Each plan generated by the KBP model required approximately 20 minutes from optimization to final dose calculation. This is significantly faster than conventional manual IMPT planning, which typically ranges from two to four hours. Although this study identified certain limitations in KBP-generated plans, it should be noted that the model-generated plan only completed a single optimization pass, and the objective list is not adjusted during optimization. In practice, such models can serve as a valuable starting point for treatment planning. Instead of beginning from scratch, planners can use the model-generated plans as a baseline and dedicate more time to addressing complex, patient-specific issues. It is important to emphasize that while KBP models can improve efficiency and consistency in treatment plans, they are not a substitute for expert clinical judgement. Ultimately, the expertise of an experienced planner remains essential for evaluating and refining model-generated plans to ensure they meet individualized clinical requirements and uphold the highest standards of care.

Previous related studies often only included patients with a single laterality or dose prescription in their models (18, 28). While this might improve the overall robustness of their model, it does not factor in more realistic deviations in clinically implemented plans. Different patients will need differing prescriptions and treatment schemes, and our models aimed to account for these differences by using patients with varying dose prescriptions. While the majority of patients received prescribed the standard 50 Gy to all target volumes (CTVs), a subset received 45 Gy, and one patient was prescribed 50.4 Gy. This variability was intentionally included to reflect the range of dose prescriptions encountered in clinical practice, with the aim of enhancing model robustness. However, it is possible that the inclusion of different prescription doses may have hindered the model’s ability to generalize by introducing noise into the learning process. In addition, outliers were not excluded in order to preserve a representative sample of our clinic’s patient population and thereby improve the generalizability of the models for future plan generation. This approach is consistent with prior reports indicating that, for a well-curated KBP model library, outlier removal has minimal impact on the quality of the resulting plans (29–31).

A limitation of this study is that patient-specific IMPT QA was not performed for the KBP-generated plans, which raises the possibility that some plans might be more challenging to deliver. Future work will incorporate delivery verification (patient-specific QA and/or log-file analysis) and plan-complexity metrics to systematically evaluate deliverability and mitigate potential library-selection bias. Another potential limitation of this study is that all 50 training cases and twenty validation cases were derived from a single institution. As a result, the models may reflect the institution-specific clinical practices, contouring styles, and machine performance which could limit generalizability of the findings. While a previous study demonstrated that a KBP model for IMPT in head and neck cancer developed at a single institution was able to generate clinically comparable plans for validation cases from an external institution (32), it is unclear whether this generalizability extends to breast and CW IMPT. Given anatomical, planning, and practice variations, it may still be advisable for each institution to create and tune their own KBP models based on their specific patient populations and workflows.

KBP models offer several advantages, including DVH estimation and significantly reduced optimization times, making it a practical tool for clinical implementation in IMPT for breast and chest wall cancers. In particular, the efficiency and automation provided by KBP may be especially beneficial for adaptive radiotherapy, where high-quality plans must be generated within a tight timeframe (13).

Future work should focus on developing and refining KBP models to further improve plan quality for unilateral (left- or right-sided) breast and chest wall treatments. Increasing the size of the training cohorts for each side-specific model may enhance performance and robustness. Despite these limitations, both models in the present study generated plans that were clinically comparable to manually generated plans in the majority of cases. Additionally, developing KBP models for IMPT of bilateral breast cancer, intentionally excluded from this study, represents an important area for future investigation. Given the strong performance of both models, KBP is likely to be highly useful for generating high-quality plans in this setting.

## Conclusions

This study evaluated the application of KBP models for IMPT in the treatment of breast or chest wall cancer with regional lymph node involvement. Two KBP models were developed: a side-specific model trained separately for each unilateral side, and a combined model trained on both sides together. Both models generated plans that were comparable to, or in some cases superior to, manually generated clinical plans, with all plans deemed clinically acceptable.

Overall, both KBP models provide robust starting points for treatment planning, offering substantial time savings and improved planning efficiency. With further validation, particularly across multiple institutions, KBP models have the potential for broader implementation, enhancing the consistency and quality of IMPT planning in clinical practice.

## Data Availability

The raw data supporting the conclusions of this article will be made available by the authors, without undue reservation.
